# An assessment of the portability of ancestry informative markers between human populations

**DOI:** 10.1186/1755-8794-2-45

**Published:** 2009-07-20

**Authors:** Sean Myles, Mark Stoneking, Nic Timpson

**Affiliations:** 1 Institute for Genomic Diversity, Cornell University, 175 Biotechnology Building, Ithaca, NY 14853-2703, USA; 2 Department of Evolutionary Genetics, Max Planck Institute for Evolutionary Anthropology, Leipzig, Germany; 3 Wellcome Trust Centre for Human Genetics, Roosevelt Drive, Oxford, OX3 7BN, UK; 4 MRC CAiTE Centre, Department of Social Medicine, University of Bristol, Oakfield House, Oakfield Grove, BS8 2BN, UK

## Abstract

**Background:**

Recent work has shown that population stratification can have confounding effects on genetic association studies and statistical methods have been developed to correct for these effects. Subsets of markers that are highly-differentiated between populations, ancestry-informative markers (AIMs), have been used to correct for population stratification. Often AIMs are discovered in one set of populations and then employed in a different set of populations. The underlying assumption in these cases is that the population under study has the same substructure as the population in which the AIMs were discovered. The present study assesses this assumption and evaluates the portability between worldwide populations of 10 SNPs found to be highly-differentiated within Britain (BritAIMs).

**Methods:**

We genotyped 10 BritAIMs in ~1000 individuals from 53 populations worldwide. We assessed the degree to which these 10 BritAIMs capture population stratification in other groups of populations by use of the Fst statistic. We used Fst values from 2750 random markers typed in the same set of individuals as an empirical distribution to which the Fst values of the 10 BritAIMs were compared.

**Results:**

Allele frequency differences between continental groups for the BritAIMs are not unusually high. This is also the case for comparisons within continental groups distantly related to Britain. However, two BritAIMs show high Fst between European populations and two BritAIMs show high Fst between populations from the Middle East. Overall the median Fst across all BritAIMs is not unusually high compared to the empirical distribution.

**Conclusion:**

We find that BritAIMs are generally not useful to distinguish between continental groups or within continental groups distantly related to Britain. Moreover, our analyses suggest that the portability of AIMs across geographical scales (e.g. between Europe and Britain) can be limited and should therefore be taken into consideration in the design and interpretation of genetic association studies.

## Background

Whole-genome association studies (GWASs) have proven extraordinarily successful in mapping loci that associate with common complex human diseases [for reviews see [1,2]]. Whereas candidate gene and linkage analyses have identified a few dozen replicable associations between genetic markers and complex diseases [3], GWASs have provided compelling evidence for more than 150 gene-disease associations since their introduction in 2006 [1]. The presence of population stratification has presented one of the main statistical challenges in GWASs. Population stratification refers to differences in allele frequencies between cases and controls related to ancestry rather than disease status. Long before technologies for GWASs were available, it was recognized that differences in ancestry between cases and controls can present a substantial confounding effect in case-control studies [4]. This is especially true in cases where disease risk differs between groups with different ancestry. For example, prostate cancer is more frequent in individuals of African ancestry compared to individuals of European ancestry [5], and previous significant genetic associations with prostate cancer become nonsignificant when correcting for these differences in ancestry [6]. The presence of population stratification can inflate false positive rates or cause reduced power and it has become standard practice to evaluate and correct for genetic ancestry in GWASs [for a review see [7]].

Currently there are two widely-used approaches for correcting for population stratification in GWASs: structured association (SA) [8] and principal components analysis (PCA) [9]. SA uses the program STRUCTURE [10] to estimate the number of sub-populations, k, and then for each individual assigns a probability of membership to each of k subpopulations. It is then tested whether allele frequencies are dependent on phenotype within each k subpopulation. PCA reduces high-dimensional data to a small number of dimensions and uses the axes of variation, or eigenvectors, from these dimensions to calculate ancestry-adjusted genotypes and phenotypes. Both of these methods rely on inferences of ancestry from genome-wide SNP data. It has been shown that accurate estimates of individual ancestry can be obtained from a subset of SNPs from genome-wide data and these are referred to as ancestry-informative markers (AIMS) [for a review see [7]]. AIMs are characterized by substantially different allele frequencies between populations and can be used to estimate the proportion of an individual's ancestry that is derived from these populations. Before running GWAS, AIMs can be used to match cases and controls, and outlier individuals whose ancestry is not typical of the population under study can be excluded [11]. The main intention for the development of sets of AIMs, however, is to provide a set of markers that effectively control for population stratification in association studies in which samples have not been typed with genome-wide SNP arrays. These sets of AIMs are designed to capture all of the necessary ancestry information required to correct for stratification in candidate gene studies, in replication studies of GWASs, or in fine-mapping studies that focus on specific genomic regions identified from GWASs.

Sets of AIMs have been developed to distinguish among continental groups [12-16]. These sets of AIMs will be useful in controlling for stratification in admixed populations especially when mapping traits that are known to differ by continental ancestry, for example skin pigmentation [17]. Most GWASs, however, have focused on samples of European ancestry and population stratification within Europe has therefore been assessed in detail [e.g. [18-20]]. From several genome-wide SNP data sets, sets of European AIMs have been developed [21-25] that distinguish stratification primarily along north-south and east-west gradients.

While European AIMs will be useful in studies that examine individuals of diverse European origin, many GWASs focus on cohorts of much more homogeneous ancestry (e.g. individuals from within a single country). It has been shown that even moderate levels of population stratification in relatively homogeneous populations can confound results in well-designed case-control studies [26,27]. For example, spurious associations due to population stratification can arise if samples are drawn from two different cities within a country [e.g. Dresden and Munich in Germany; [18]] and can even appear in genetic isolates like the Icelandic [28] and Finnish populations [29]. Despite these warnings, association studies that do not properly correct for population structure continue to be published [e.g. [30]].

Studies that do incorporate a correction for the confounding effects of population structure are to be commended. However, the correction for population structure is only as good as the markers chosen for the correction. In general, the precondition for the use of a set of AIMs is that the population under study has the same substructure as the population in which the AIMs were discovered. In several recent association studies, this precondition has been left unevaluated and potentially uninformative AIMs have been used to correct for population stratification. For example, Sulem et al. [31] tested for the presence of population stratification in Iceland with a set of AIMs that distinguish between European populations [24]. To correct for population stratification in association studies in Asian populations, SNPs with high Fst between Asians and other continental groups have been employed [32,33]. Similarly, correcting for population structure in Caucasians, Hu et al. [34] use 38 SNPs that are highly differentiated between continental groups. These studies demonstrate that the underlying assumption that AIMs are largely portable across geographical scales is pervasive.

To assess the portability of AIMs between populations and across geographic scales, we genotyped 10 SNPs found to be highly-differentiated within Britain (BritAIMs) in ~1000 individuals from 53 populations worldwide. Although these 10 BritAIMs do not constitute a complete set of AIMs that fully capture population structure within Britain, they are nevertheless useful for evaluating the portability of AIMs across geographic scales. We evaluated the usefulness of these BritAIMs as AIMs between and within different continents by comparing Fst values for the BritAIMs to Fst values from 2750 random markers typed in the same set of worldwide samples. Our results suggest that AIMs have limited portability between human populations and that caution is warranted in the use of AIMs discovered in a population whose substructure does not match the population in which they are being employed.

## Methods

We selected 13 SNPs identified as "highly-differentiated" within Britain from a data set of ~500,000 SNPs typed in ~16,000 British individuals [see Table 1; [35]]. These highly-differentiated SNPs had the lowest P values from a χ^2 ^test of allele frequency difference between 12 geographic regions of Britain defined by postcode. We genotyped these 13 SNPs in the CEPH-HGDP Panel [36]. The CEPH-HGDP panel includes 952 individuals from 53 populations after the removal of atypical and related individuals [37]. Genotypes were generated by KBioscience using a competitive allele-specific PCR SNP genotyping system [38]. Cluster plots were analysed visually and the following 3 SNPs were removed. SNP rs6644913 was removed because it maps to the X chromosome; we restricted our analyses to the autosomes because Fst values from the sex chromosomes are not comparable to autosomal Fst values. SNP rs3873375 was removed because it maps to multiple genomic regions and shows 4 distinct genotype clusters. Finally, the genotyping of SNP rs1042712 failed completely. As independent verification of the 10 remaining SNPs, our genotype data were compared to genotypes generated from 67 individuals of diverse ancestry from the CEPH-HGDP panel in the laboratory of MS using the Affymetrix GeneChip 500K Mapping Array Set (unpublished data). One SNP, rs12797951, showed substantial discordance between our genotypes and the genotypes from the Affymetrix platform (data not shown). We therefore retrieved genotype data for this SNP from the ~650,000 SNPs typed in the CEPH-HGDP using Illumina HumanHap650K Beadchips [39]. The Illumina and Affy data were consistent for rs12797951 and we therefore used the data from Li et al. [39] for SNP rs12797951. The final set of 10 SNPs are the British ancestry-informative markers (BritAIMs) presented in Table 1.

**Table 1:** Fst values of the 10 BritAIMs and the associated *P *values.

	Global		Europe		Middle East		Africa		Central South Asia		East Asia		Oceania		America	
BritAIMs	Fst	*P*	Fst	*P*	Fst	*P*	Fst	*P*	Fst	*P*	Fst	*P*	Fst	*P*	Fst	*P*
rs7696175	0.074	0.551	0.129	**0.000**	0.002	0.840	0.052	0.469	0.011	0.748	0.018	0.802	0.067	0.439	0.118	0.333
rs1460133	0.035	0.842	0.062	**0.043**	0.027	0.286	0	1	0.022	0.502	0.010	0.916	NA	NA	0.042	0.695
rs9378805	0.115	0.321	0.030	0.280	0.043	0.136	0.019	0.796	0.020	0.554	0.016	0.831	0.072	0.424	0.027	0.754
rs11790408	0.067	0.594	0.043	0.141	0.088	**0.022**	0.028	0.714	0.046	0.169	0.009	0.920	0.015	0.709	0.108	0.370
rs12295525	0.068	0.591	0.034	0.226	0.008	0.666	0.047	0.513	0.024	0.454	0.017	0.809	NA	NA	0.021	0.820
rs12797951	0.106	0.365	0	1	0.105	**0.015**	0.037	0.611	0.053	0.116	0.061	0.136	NA	NA	0.175	0.167
rs10774241	0.103	0.383	0	1	0	1	0.090	0.226	0.024	0.470	0.029	0.580	0.004	0.760	0.139	0.247
rs17449560	0.036	0.835	0.017	0.520	0.043	0.135	0.112	0.146	0.003	0.909	0.017	0.814	0.199	0.210	0.061	0.581
rs3760843	0.033	0.855	0.024	0.369	0	1	0.073	0.320	0.014	0.665	0.021	0.747	0.144	0.295	0.139	0.247
rs2143877	0.078	0.529	0.046	0.117	0.011	0.585	0.053	0.460	0.004	0.892	0.015	0.857	0.005	0.754	0.038	0.714

Global Fst was calculated among the 7 continental regions listed as column titles. *P *values were generated by comparison to an empirical distribution of 2750 markers (see Methods). Significant *P *values (*P *< 0.05) are in bold.

Genotype calls were made by visual inspection. None of the 10 SNPs were out of Hardy-Weinberg equilibrium after Bonferroni correction for multiple comparisons (53 populations × 10 SNPs = 530 comparisons). In the 7 cases of significant (*P *< 0.05 without Bonferroni correction) deviation from Hardy-Weinberg expectations for a SNP in a population, cluster plots were re-evaluated and no data were removed. The amount of missing data per SNP ranged from 0% – 6.5% with a mean of 3.1%. These data are accessible by request to the corresponding author or from the CEPH database [40].

Fst was calculated according to equation 10 in Weir and Cockerham [41]. Negative Fst values were set to 0. "Global Fst" for each of the 10 SNPs was calculated as the degree of differentiation among the 7 geographic regions represented in the CEPH-HGDP panel. Results remain unchanged when global Fst was calculated as the differentiation among all 53 populations rather than the 7 regions. We compared our observed Fst values for the 10 BritAIMs to an empirical Fst distribution from 2750 autosomal markers (2540 SNPs [42] and 210 indels [43]) typed in 927 individuals from the CEPH-HGDP panel. In cases where the same allele was fixed in all populations being compared, the SNP was considered non-informative and no Fst value was assigned. This resulted in different numbers of observations for the different empirical distributions with a minimum 2286 SNPs making up the empirical Fst distribution within Oceania. To allow for an unbiased comparison to the empirical distribution, Fst for the 10 BritAIMs was calculated from the same set of 927 individuals from which the empirical Fst distribution was calculated. For each BritAIM, a *P *value was calculated as the proportion of Fst values from the empirical distribution that were ≥ the observed Fst value. We use *P *< 0.05 as our threshold for "significance". It should be noted that "significant" therefore describes a value only in relation to the empirical distribution.

## Results

To assess the portability of AIMs, we used the Fst statistic [44] to measure the degree of genetic differentiation within and between continental groups of ten BritAIMs, i.e. SNPs identified as "highly-differentiated" within Britain [35]. Fst is a commonly employed and useful measure of allele frequency difference between populations and takes on values ranging from 0 (no difference) to 1 (fixed difference). SNPs with high Fst values are highly differentiated between populations and are therefore informative about population structure and are useful as AIMs [e.g. [23,45]]. The list of 10 BritAIMS is presented in Table 1 along with Fst values and associated *P *values for the among-continent (i.e. global) and within-continent comparisons.

**Figure 1:**
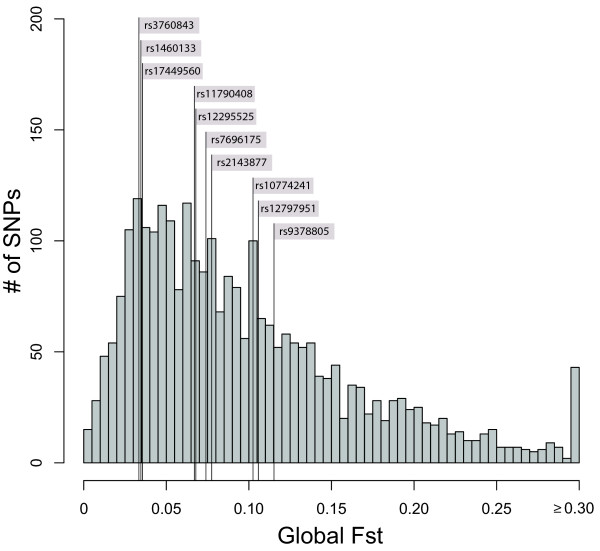
**Global Fst distribution for 2750 random markers**. The global Fst values of the 10 BritAIMs are indicated by vertical lines.

**Figure 2:**
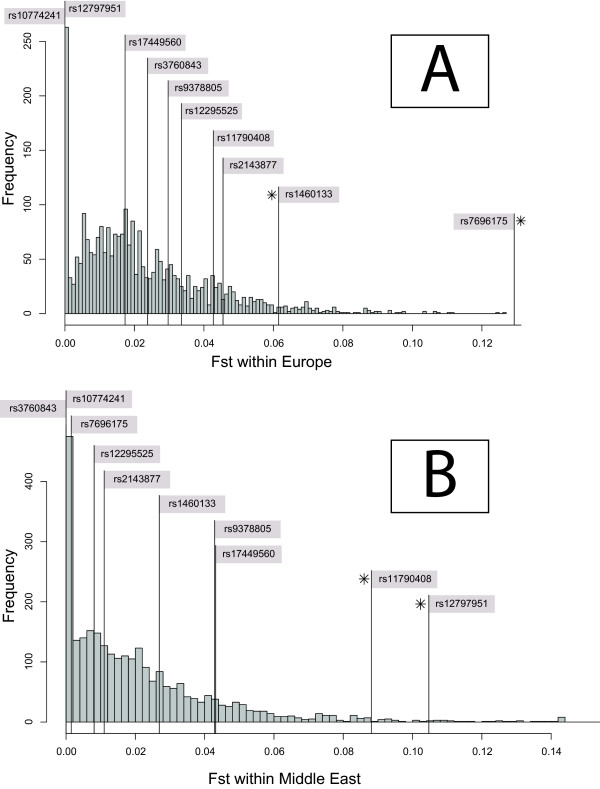
**Fst values of the 2750 random markers within Europe (A) and within the Middle East (B)**. The Fst values of the 10 BritAIMs are indicated by vertical lines. BritAIMs that lie within the top 5% of the empirical distribution are highlighted with asterisks.

We first tested whether the 10 BritAIMs were highly differentiated among the 7 continental groups of the CEPH-HGDP panel by comparing global Fst values of the BritAIMs to the empirical distribution of global Fst values from 2750 random markers. None of the 10 BritAIMs have significantly high global Fst values (Figure 1, Table 1). We assessed population differentiation on a finer geographical scale by calculating Fst within continents. The *P *values for each of these comparisons are presented in Table 1. Only 4 BritAIMs showed significantly high Fst values (*P *< 0.05) in the within-continent analyses and these are highlighted in bold in Table 1: two BritAIMs (rs7696175, rs1460133) showed significantly high Fst within Europe and two were significant within the Middle East (rs11790408, rs12797951). Figure 2 displays the positions of the BritAIMs in the empirical Fst distributions of Europe and the Middle East.

To test whether BritAIMs are highly differentiated as a group, we compared the median Fst of the 10 BritAIMs to a distribution of median Fst values from 10 SNPs sampled at random 10,000 times from the empirical distribution. This allows an assessment of how differentiated the 10 BritAIMs are compared to the expectation at random. At the worldwide scale, the median global Fst of the 10 BritAIMs does not differ significantly from the expectation generated from 10,000 random samples (Fst = 0.071, *P *= 0.692). However, the comparisons within each continental group revealed that the median Fst of the BritAIMs is significantly high within Europe (median Fst = 0.0316, *P *= 0.039; Table 2).

**Table 2:** Median Fst of the 10 BritAIMs within each continent and the associated *P *values

**Continental Group**	**Median Fst**	***P *value**
Europe	0.0316	**0.039**
Central South Asia	0.021	0.574
Africa	0.0492	0.49
Middle East	0.01895	0.302
East Asia	0.017	0.992
America	0.08465	0.414
Oceania	0.067	0.362

*P *values generated by random sampling from the empirical distribution (see Methods). Significant *P *values (*P *< 0.05) are in bold.

**Figure 3:**
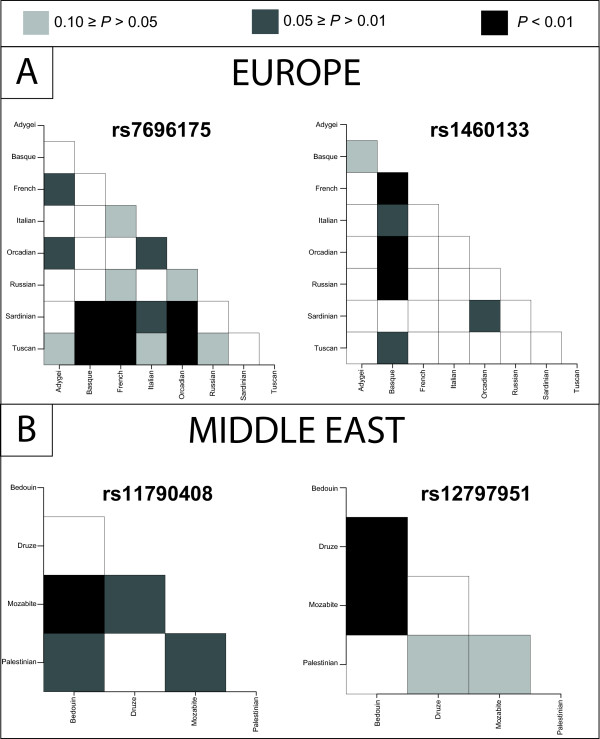
**Levels of population differentiation for 2 BritAIMs with significantly high Fst within Europe (A) and 2 BritAIMs with significantly high Fst within the Middle East (B)**. Each box in each matrix represents a population pairwise Fst comparison. The shaded boxes in the matrices show which pairwise Fst values are significant compared to the empirical distribution at three *P *value thresholds (see the *P *value legend).

**Figure 4:**
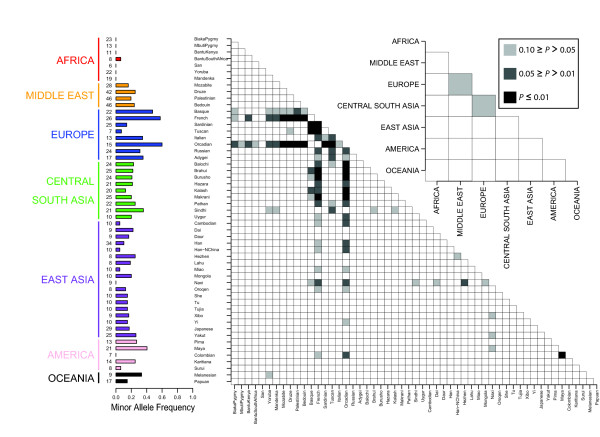
**Worldwide allele frequencies and population differentiation for rs7696175, the most highly differentiated BritAIM within Europe**. The vertical bar chart displays the minor allele frequency in each of the populations represented in the CEPH-HGDP panel with sample sizes (number of individuals) on the left. The shaded boxes in the 53 × 53 and 7 × 7 matrices show which pairwise Fst values are significant compared to the empirical distribution at three *P *value thresholds (see the boxed-in *P *value legend) for the population by population comparison and the continent by continent comparison, respectively.

Fst calculations within a continent summarize the degree of allele frequency differentiation among all populations within a continent and can be driven to high values by single outlier populations. Therefore, we investigated the four BritAIMs with significantly high Fst values in more detail by calculating Fst for each pairwise comparison between populations within a continent. Figure 3 shows how these population pairwise Fst values compare to the corresponding Fst values from the empirical distribution. Finally, Figure 4 provides a view of worldwide allele frequencies and population differentiation for rs7696175, the most highly-differentiated BritAIM within Europe. Similar plots of worldwide allele frequencies and population differentiation are provided for each BritAIM in Additional File 1.

## Discussion

The presence of population stratification is a potential source of false positives, and thus of spurious associations, in disease association studies. Recently, a number of studies have identified AIMs and have recommended their use to control for population stratification in association studies [12,13,21,22,25]. The sets of AIMs identified to date show large allele frequency differences either between continental groups (e.g. Africans and Europeans) or between populations within a continent (e.g. Europe). However, many association studies are conducted in relatively homogeneous populations. The medical genetics literature provides numerous examples where AIMs discovered at one geographic scale are used to correct for population stratification at finer geographic scales [e.g. [31-34]]. It remains unclear, however, whether AIMs identified as informative within Europe, for example, will also prove useful at finer geographical scales (e.g. within Britain). The present study takes a first step in addressing this issue by examining the portability of AIMs between populations and across geographic scales.

The 10 SNPs identified as highly-differentiated within Britain (BritAIMs) are not highly differentiated on a worldwide scale: none of the global Fst values for the BritAIMs lie in the upper tail of the empirical global Fst distribution (Figure 1). The median global Fst of the 10 BritAIMs is also not unusually high compared to the expectation at random (*P *= 0.692). Thus, AIMs identified at a fine geographic scale (i.e. within Britain) are not informative on a worldwide scale. This result was foreseeable since there is no a priori reason for expecting SNPs that differ dramatically in allele frequency within Britain to differ dramatically among continental groups.

Within continents, only 4 of the 10 BritAIMs have significantly high Fst values (Figure 2). These 4 BritAIMs are found within the top 5% of the empirical distributions from Europe and the Middle East. These two continental groups are assumed to be more closely related to Britons than the other continental groups included in the present study, and this result therefore suggests that some AIMs may be portable within a restricted geographic range. When the patterns of population differentiation for these 4 BritAIMs are examined in more detail, it is clear that the signal from rs1460133 is derived almost exclusively from the Basque who differ significantly from most of the other European populations for this SNP (Figure 3). Thus, while rs1460133 may be an informative marker for Basque ancestry, it does not show the dramatic gradient of allele frequency across the continent that is characteristic of other European AIMs [24]. It is noteworthy that the median Fst for the 10 BritAIMs is significantly high within Europe (*P *= 0.039), but not within the other continental groups (Table 2). This observation also supports the notion that the BritAIMs as a set are at least somewhat ancestry informative across European populations.

Figure 4 provides a detailed view of the allele frequencies and population differentiation of the BritAIM that shows the highest Fst value within Europe, rs7696175. From the 53 × 53 matrix in Figure 4, it can be seen that the high Fst values for rs7696175 are not restricted to population pairwise comparisons within Europe: the French and Orcadians, for example, have substantially higher minor allele frequencies than several populations from the Middle East and Central South Asia. Similar plots are available for the remaining 9 BritAIMs (Additional File 1) in which it can be seen that the patterns of population differentiation are extremely varied across SNPs: many population-pairwise Fst values lie within the top 5% and even the top 1% of the empirical distribution. Thus the BritAIMs may be useful as AIMs in other groups of populations, but the patterns are often not systematic and their effectiveness in other samples would be difficult to predict *a priori*.

Previous studies have provided some evidence that AIMs may be portable between human populations. For example, microsatellite markers that are ancestry informative in one population are generally informative in others [45]. Also, genomic regions showing large allele frequency differences between one set of continental groups are likely to be highly-differentiated between other continental groups [46]. However, more recent studies that focus on the portability of AIMs across continental groups provide evidence against this notion. For example, Campbell et al. [47] previously noted that the use of 67 AIMs that were discovered to distinguish between African and European ancestry did not vary sufficiently among Europeans to allow detection of stratification. Similarly, Paschou et al. [48] found that SNPs chosen for ancestry inference in one continent perform no better than random SNPs in inferring ancestry in other continents. These studies have focused, however, on the portability of AIMs across broad geographic scales (i.e. between continental groups) and their conclusions have limited applicability to the design of association studies which usually focus on a more refined geographic scale.

Recently, Heath et al. [18] used PCA to assess population structure in ~6000 Europeans genotyped for ~130,000 SNPs and found that 5 of the 10 genomic regions containing the BritAIMs examined here were significantly associated with PC1 or PC2. It is worth noting that the two BritAIMs for which genotyping failed in the present study were in regions significantly correlated with PC2, and that 4 of the 5 remaining genomic regions containing BritAIMs neared significant correlation with PC1 or PC2 [18]. These data suggest that, while AIMs discovered in a broad panel of Europeans may not perfectly capture ancestry information within Britain, there is substantial overlap among ancestry-informative genomic regions between the two geographic scales.

Local, geographically-restricted, natural selection at a locus generates large allele frequency differences between populations [49,50]. Thus, AIMs are enriched in genomic regions that have been targeted by positive selection and are therefore likely to be in LD with adaptive functional alleles. Viewed from this evolutionary perspective, our finding that BritAIMs are not unusually differentiated between continental groups is not surprising: selection pressures that have generated allele frequency differences within Britain are unlikely to be shared across continental groups because local cultural and physical environments differ drastically at the continental scale. However, the BritAIMs' sharp gradients of allele frequencies across Britain are likely to have been caused by selection pressures shared by other European populations. For example, one BritAIM (rs1042712) is found near the lactase gene which shows a sharp gradient across Europe due to the action of positive selection for lactose tolerance [51]. Thus, the portability of AIMs between populations will depend in part on the extent to which selection pressures have been shared between the populations. Without extensive population genetic analyses, this criterion will be difficult to evaluate.

## Conclusion

The assumption that AIMs are portable across geographic scales is pervasive [31-34]. The data presented here suggest that there is an inevitable loss of power to detect population stratification when AIMs discovered in one population are used in another population. Practically, the assumption that the substructure of the population under study is adequately similar to the substructure of the population in which the AIMs were discovered is often difficult to evaluate. The present analyses suggest the portability of AIMs is limited and that claims of association between genetic variants and phenotypes should be interpreted in accordance with the suitability of the selected AIMs used to correct for population stratification. As association analyses become increasingly common in populations for which genome-wide genotype data is sparse, we anticipate that this cautionary note will become increasingly important.

## Competing interests

The authors declare that they have no competing interests.

## Authors' contributions

SM, MS and NT designed the study; SM performed statistical analyses; SM, MS and NT wrote the manuscript.

## Pre-publication history

The pre-publication history for this paper can be accessed here:



## Supplementary Material

Additional file 1 **Worldwide risk allele frequencies and population differentiation for the 10 BritAIMs**. The dbSNP ID is found at the top of each figure. Minor allele frequencies are displayed in the vertical bar chart with sample size in number of individuals to the left. Each box in the 53 × 53 and 7 × 7 matrices represents a pairwise Fst comparison between populations and geographic regions, respectively. The shaded boxes in the matrices indicate which pairwise Fst values are significant compared to the empirical distribution at three *P *value thresholds (see the boxed-in *P *value legend of Figure 4). Click here for file

## References

[B1] Altshuler D, Daly MJ, Lander ES (2008). Genetic Mapping in Human Disease. Science.

[B2] McCarthy MI, Abecasis GR, Cardon LR, Goldstein DB, Little J, Ioannidis JPA, Hirschhorn JN (2008). Genome-wide association studies for complex traits: consensus, uncertainty and challenges. Nat Rev Genet.

[B3] Lohmueller KE, Pearce CL, Pike M, Lander ES, Hirschhorn JN (2003). Meta-analysis of genetic association studies supports a contribution of common variants to susceptibility to common disease. Nat Genet.

[B4] Knowler WC, Williams RC, Pettitt DJ, Steinberg AG (1988). Gm3;5,13,14 and type 2 diabetes mellitus: an association in American Indians with genetic admixture. Am J Hum Genet.

[B5] Bunker CH, Patrick AL, Konety BR, Dhir R, Brufsky AM, Vivas CA, Becich MJ, Trump DL, Kuller LH (2002). High prevalence of screening-detected prostate cancer among Afro-Caribbeans: the Tobago Prostate Cancer Survey. Cancer Epidemiol Biomarkers Prev.

[B6] Kittles RA, Chen W, Panguluri RK, Ahaghotu C, Jackson A, Adebamowo CA, Griffin R, Williams T, Ukoli F, Adams-Campbell L (2002). CYP3A4-V and prostate cancer in African Americans: causal or confounding association because of population stratification?. Hum Genet.

[B7] Tian C, Gregersen PK, Seldin MF (2008). Accounting for ancestry: population substructure and genome-wide association studies. Hum Mol Genet.

[B8] Pritchard JK, Stephens M, Rosenberg NA, Donnelly P (2000). Association mapping in structured populations. Am J Hum Genet.

[B9] Price AL, Patterson NJ, Plenge RM, Weinblatt ME, Shadick NA, Reich D (2006). Principal components analysis corrects for stratification in genome-wide association studies. Nat Genet.

[B10] Pritchard JK, Stephens M, Donnelly P (2000). Inference of Population Structure Using Multilocus Genotype Data. Genetics.

[B11] Hinds DA, Stokowski RP, Patil N, Konvicka K, Kershenobich D, Cox DR, Ballinger DG (2004). Matching strategies for genetic association studies in structured populations. Am J Hum Genet.

[B12] Kosoy R, Nassir R, Tian C, White PA, Butler LM, Silva G, Kittles R, Alarcon-Riquelme ME, Gregersen PK, Belmont JW (2008). Ancestry informative marker sets for determining continental origin and admixture proportions in common populations in America. Hum Mutat.

[B13] Halder I, Shriver M, Thomas M, Fernandez JR, Frudakis T (2008). A panel of ancestry informative markers for estimating individual biogeographical ancestry and admixture from four continents: utility and applications. Hum Mutat.

[B14] Yang N, Li H, Criswell LA, Gregersen PK, Alarcon-Riquelme ME, Kittles R, Shigeta R, Silva G, Patel PI, Belmont JW (2005). Examination of ancestry and ethnic affiliation using highly informative diallelic DNA markers: application to diverse and admixed populations and implications for clinical epidemiology and forensic medicine. Hum Genet.

[B15] Enoch MA, Shen PH, Xu K, Hodgkinson C, Goldman D (2006). Using ancestry-informative markers to define populations and detect population stratification. J Psychopharmacol.

[B16] Lao O, Duijn Kv, Kersbergen P, Knijff Pd, Kayser M (2006). Proportioning Whole-Genome Single-Nucleotide-Polymorphism Diversity for the Identification of Geographic Population Structure and Genetic Ancestry. Am J Hum Genet.

[B17] Hoggart CJ, Parra EJ, Shriver MD, Bonilla C, Kittles RA, Clayton DG, McKeigue PM (2003). Control of confounding of genetic associations in stratified populations. Am J Hum Genet.

[B18] Heath SC, Gut IG, Brennan P, McKay JD, Bencko V, Fabianova E, Foretova L, Georges M, Janout V, Kabesch M (2008). Investigation of the fine structure of European populations with applications to disease association studies. Eur J Hum Genet.

[B19] Novembre J, Johnson T, Bryc K, Kutalik Z, Boyko AR, Auton A, Indap A, King KS, Bergmann S, Nelson MR (2008). Genes mirror geography within Europe. Nature.

[B20] Lao O, Lu TT, Nothnagel M, Junge O, Freitag-Wolf S, Caliebe A, Balascakova M, Bertranpetit J, Bindoff LA, Comas D (2008). Correlation between Genetic and Geographic Structure in Europe. Curr Biol.

[B21] Tian C, Plenge RM, Ransom M, Lee A, Villoslada P, Selmi C, Klareskog L, Pulver AE, Qi L, Gregersen PK (2008). Analysis and application of European genetic substructure using 300 K SNP information. PLoS Genet.

[B22] Price AL, Butler J, Patterson N, Capelli C, Pascali VL, Scarnicci F, Ruiz-Linares A, Groop L, Saetta AA, Korkolopoulou P (2008). Discerning the ancestry of European Americans in genetic association studies. PLoS Genet.

[B23] Bauchet M, McEvoy B, Pearson LN, Quillen EE, Sarkisian T, Hovhannesyan K, Deka R, Bradley DG, Shriver MD (2007). Measuring European Population Stratification with Microarray Genotype Data. Am J Hum Genet.

[B24] Seldin MF, Shigeta R, Villoslada P, Selmi C, Tuomilehto J, Silva G, Belmont JW, Klareskog L, Gregersen PK (2006). European Population Substructure: Clustering of Northern and Southern Populations. PLoS Genet.

[B25] Paschou P, Drineas P, Lewis J, Nievergelt CM, Nickerson DA, Smith JD, Ridker PM, Chasman DI, Krauss RM, Ziv E (2008). Tracing Sub-Structure in the European American Population with PCA-Informative Markers. PLoS Genet.

[B26] Marchini J, Cardon LR, Phillips MS, Donnelly P (2004). The effects of human population structure on large genetic association studies. Nat Genet.

[B27] Freedman ML, Reich D, Penney KL, McDonald GJ, Mignault AA, Patterson N, Gabriel SB, Topol EJ, Smoller JW, Pato CN (2004). Assessing the impact of population stratification on genetic association studies. Nat Genet.

[B28] Helgason A, Yngvadottir B, Hrafnkelsson B, Gulcher J, Stefansson K (2005). An Icelandic example of the impact of population structure on association studies. Nat Genet.

[B29] Jakkula E, Rehnström K, Varilo T, Pietiläinen OPH, Paunio T, Pedersen NL, deFaire U, Järvelin M-R, Saharinen J, Freimer N (2008). The Genome-wide Patterns of Variation Expose Significant Substructure in a Founder Population. Am J Hum Genet.

[B30] Pfeufer A, Sanna S, Arking DE, Muller M, Gateva V, Fuchsberger C, Ehret GB, Orru M, Pattaro C, Kottgen A (2009). Common variants at ten loci modulate the QT interval duration in the QTSCD Study. Nat Genet.

[B31] Sulem P, Gudbjartsson DF, Stacey SN, Helgason A, Rafnar T, Magnusson KP, Manolescu A, Karason A, Palsson A, Thorleifsson G (2007). Genetic determinants of hair, eye and skin pigmentation in Europeans. Nat Genet.

[B32] Fujimoto A, Kimura R, Ohashi J, Omi K, Yuliwulandari R, Batubara L, Mustofa MS, Samakkarn U, Settheetham-Ishida W, Ishida T (2008). A scan for genetic determinants of human hair morphology: EDAR is associated with Asian hair thickness. Hum Mol Genet.

[B33] Fujimoto A, Ohashi J, Nishida N, Miyagawa T, Morishita Y, Tsunoda T, Kimura R, Tokunaga K (2008). A replication study confirmed the EDAR gene to be a major contributor to population differentiation regarding head hair thickness in Asia. Hum Genet.

[B34] Hu Y, Li L, Seidelmann SB, Timur AA, Shen PH, Driscoll DJ, Wang QK (2008). Identification of Association of Common AGGF1 Variants with Susceptibility for Klippel-Trenaunay Syndrome Using the Structure Association Program. Ann Hum Genet.

[B35] The Wellcome Trust Case Control Consortium (2007). Genome-wide association study of 14,000 cases of seven common diseases and 3,000 shared controls. Nature.

[B36] Cann HM, de Toma C, Cazes L, Legrand MF, Morel V, Piouffre L, Bodmer J, Bodmer WF, Bonne-Tamir B, Cambon-Thomsen A (2002). A human genome diversity cell line panel. Science.

[B37] Rosenberg NA (2006). Standardized Subsets of the HGDP-CEPH Human Genome Diversity Cell Line Panel, Accounting for Atypical and Duplicated Samples and Pairs of Close Relatives. Ann Hum Genet.

[B38] KBiosciences http://www.kbioscience.co.uk/genotyping/genotyping_chemistry.html.

[B39] Li JZ, Absher DM, Tang H, Southwick AM, Casto AM, Ramachandran S, Cann HM, Barsh GS, Feldman M, Cavalli-Sforza LL (2008). Worldwide Human Relationships Inferred from Genome-Wide Patterns of Variation. Science.

[B40] CEPH http://www.cephb.fr/cephdb/.

[B41] Weir BS, Cockerham CC (1984). Estimating F-statistics for the analysis of population structure. Evolution.

[B42] Conrad DF, Jakobsson M, Coop G, Wen X, Wall JD, Rosenberg NA, Pritchard JK (2006). A worldwide survey of haplotype variation and linkage disequilibrium in the human genome. Nat Genet.

[B43] Rosenberg NA, Mahajan S, Ramachandran S, Zhao C, Pritchard JK, Feldman MW (2005). Clines, Clusters, and the Effect of Study Design on the Inference of Human Population Structure. PLoS Genet.

[B44] Wright S (1969). The theory of gene frequencies.

[B45] Rosenberg NA, Li LM, Ward R, Pritchard JK (2003). Informativeness of genetic markers for inference of ancestry. Am J Hum Genet.

[B46] Myles S, Tang K, Somel M, Green RE, Kelso J, Stoneking M (2008). Identification and analysis of high Fst regions from genome-wide SNP data from three human populations. Ann Hum Genet.

[B47] Campbell CD, Ogburn EL, Lunetta KL, Lyon HN, Freedman ML, Groop LC, Altshuler D, Ardlie KG, Hirschhorn JN (2005). Demonstrating stratification in a European American population. Nat Genet.

[B48] Paschou P, Ziv E, Burchard EG, Choudhry S, Rodriguez-Cintron W, Mahoney MW, Drineas P (2007). PCA-Correlated SNPs for Structure Identification in Worldwide Human Populations. PLoS Genet.

[B49] Beaumont MA, Balding DJ (2004). Identifying adaptive genetic divergence among populations from genome scans. Mol Ecol.

[B50] Pollinger JP, Bustamante CD, Fledel-Alon A, Schmutz S, Gray MM, Wayne RK (2005). Selective sweep mapping of genes with large phenotypic effects. Genome Res.

[B51] Bersaglieri T, Sabeti PC, Patterson N, Vanderploeg T, Schaffner SF, Drake JA, Rhodes M, Reich DE, Hirschhorn JN (2004). Genetic signatures of strong recent positive selection at the lactase gene. Am J Hum Genet.

