# Supplementary figures and images for: An assessment of the portability of ancestry informative markers between human populations
Source: BMC Med Genomics. 2009 Jul 20;2:45. doi: 10.1186/1755-8794-2-45 (PMC2719660; doi:10.1186/1755-8794-2-45)

rs7696175

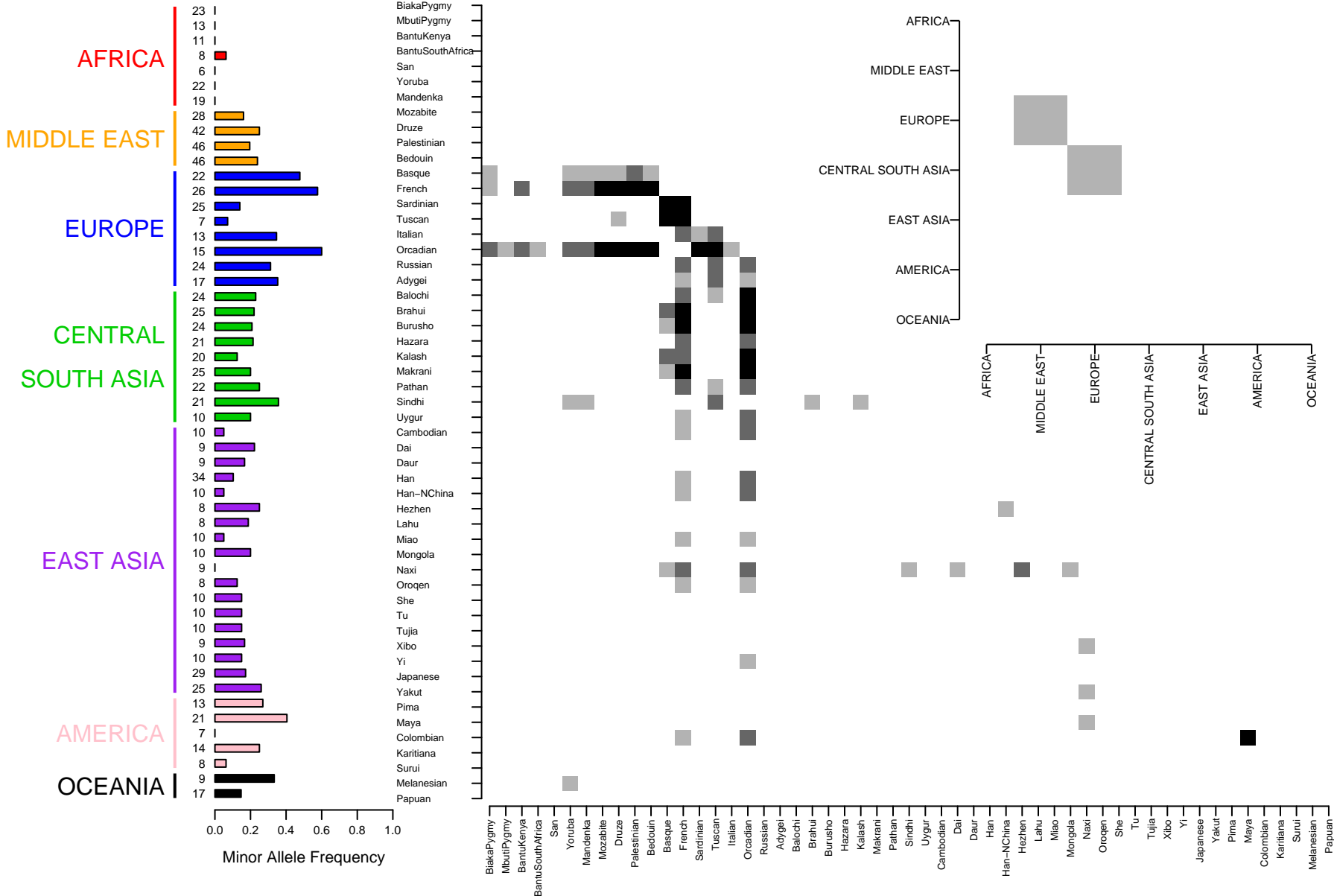

rs1460133

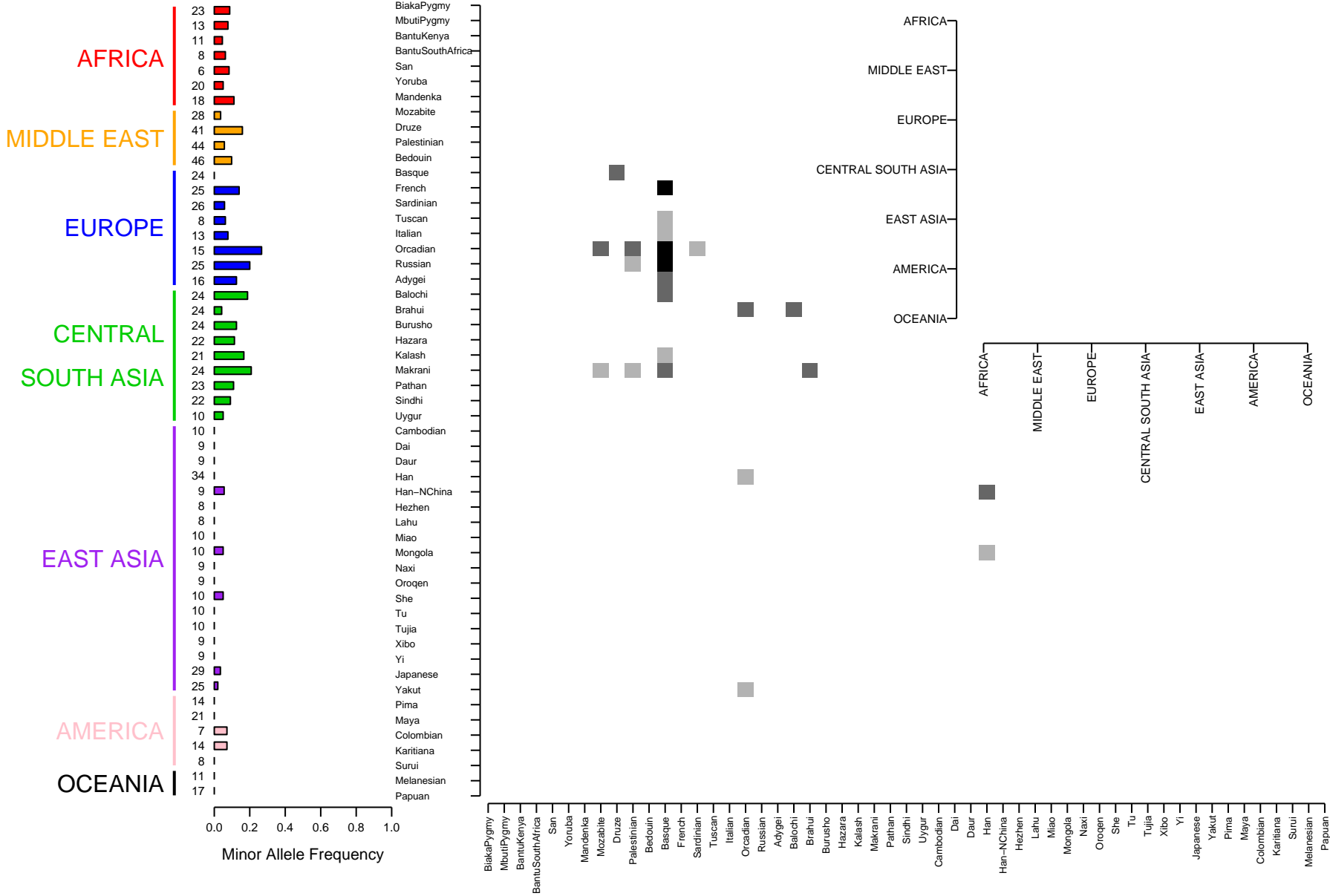

rs9378805

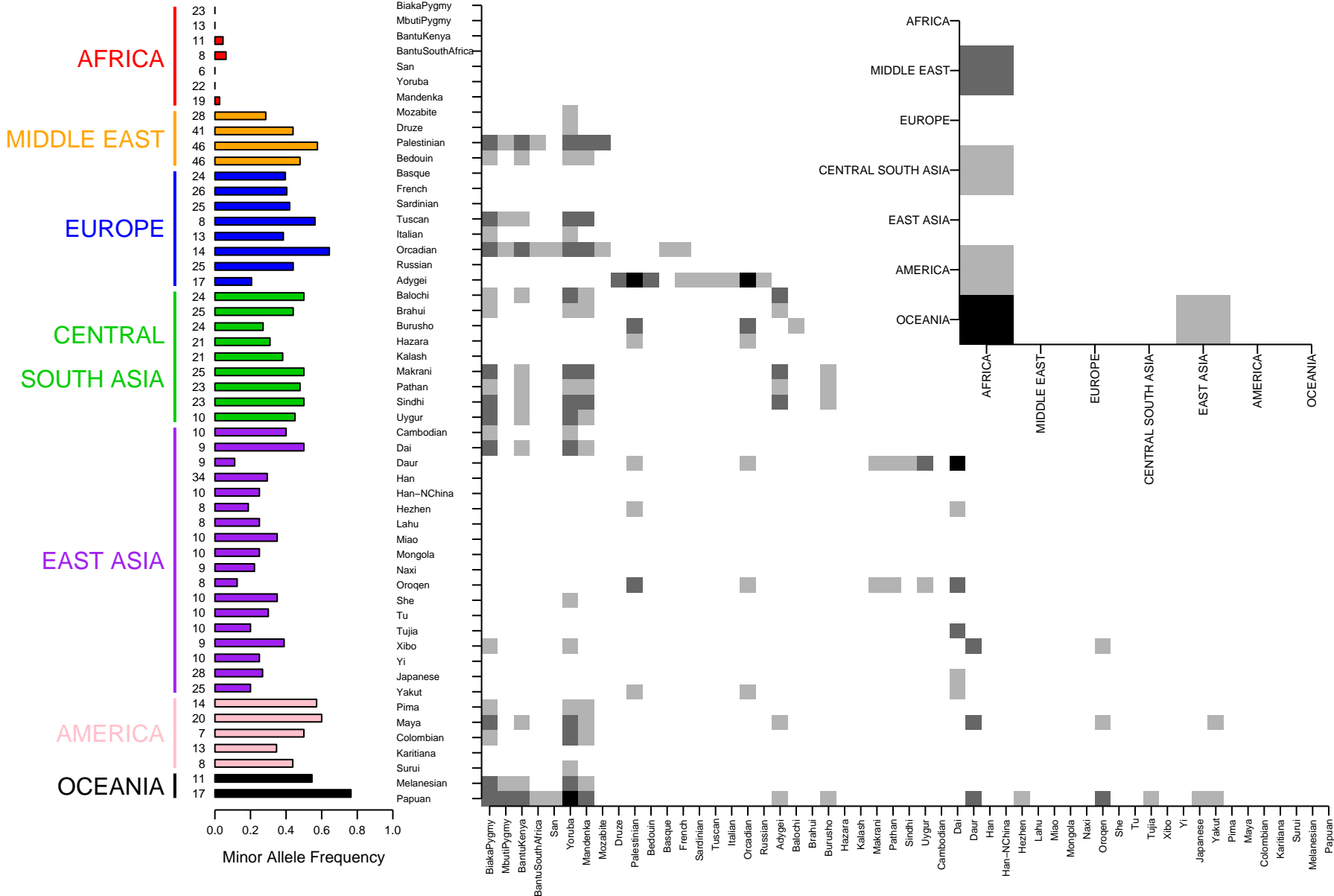

rs11790408

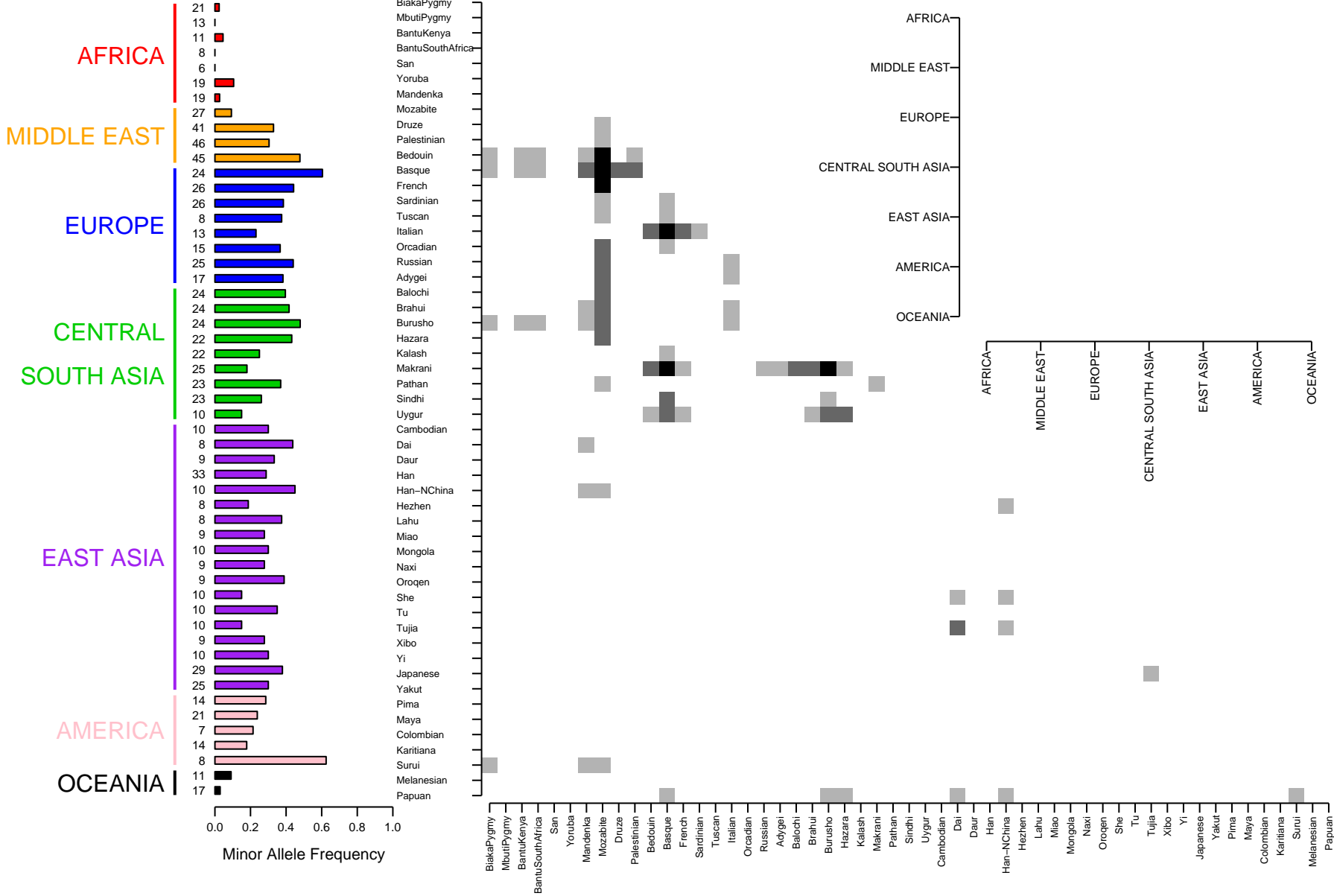

rs12295525

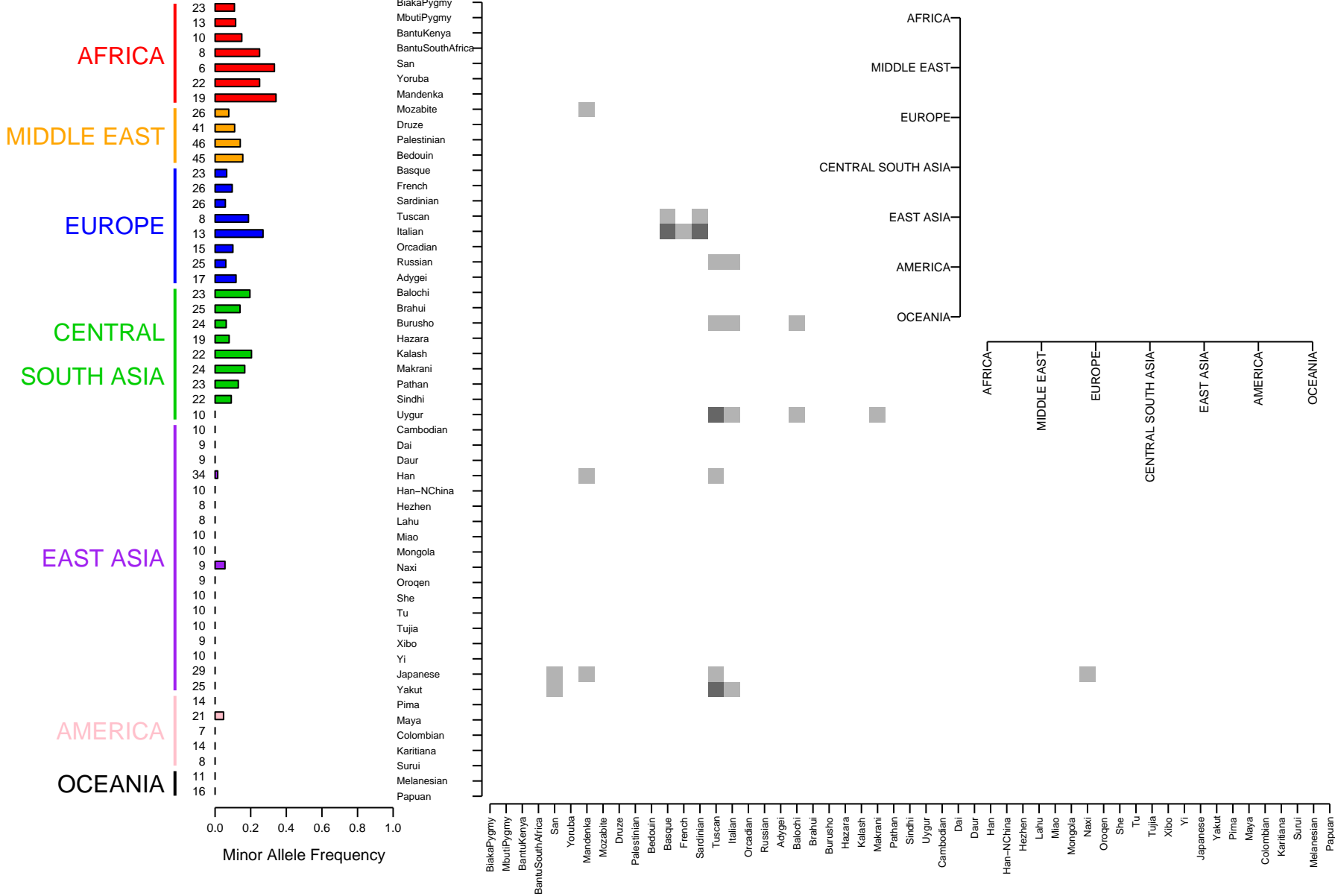

rs12797951

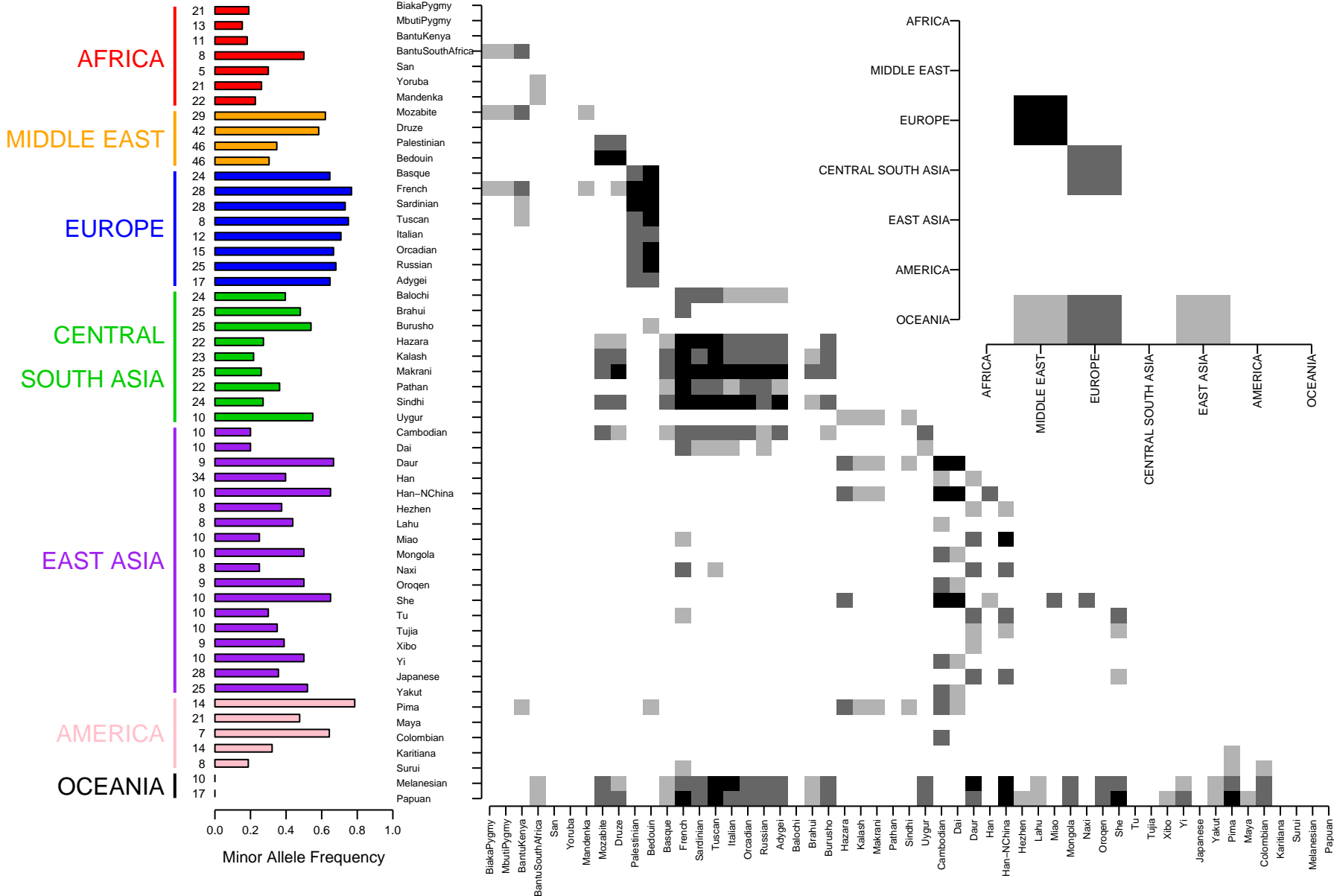

rs10774241

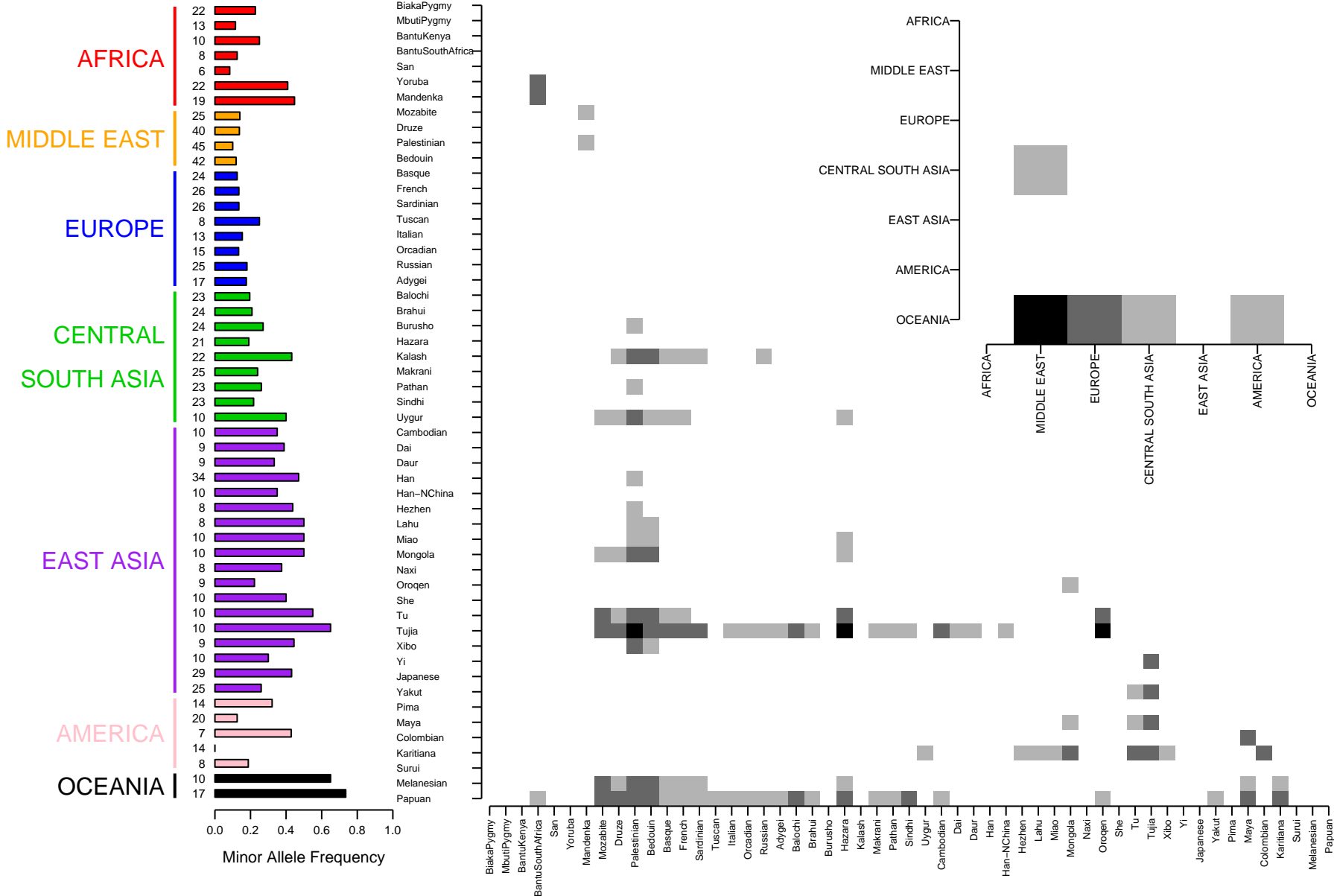

rs17449560

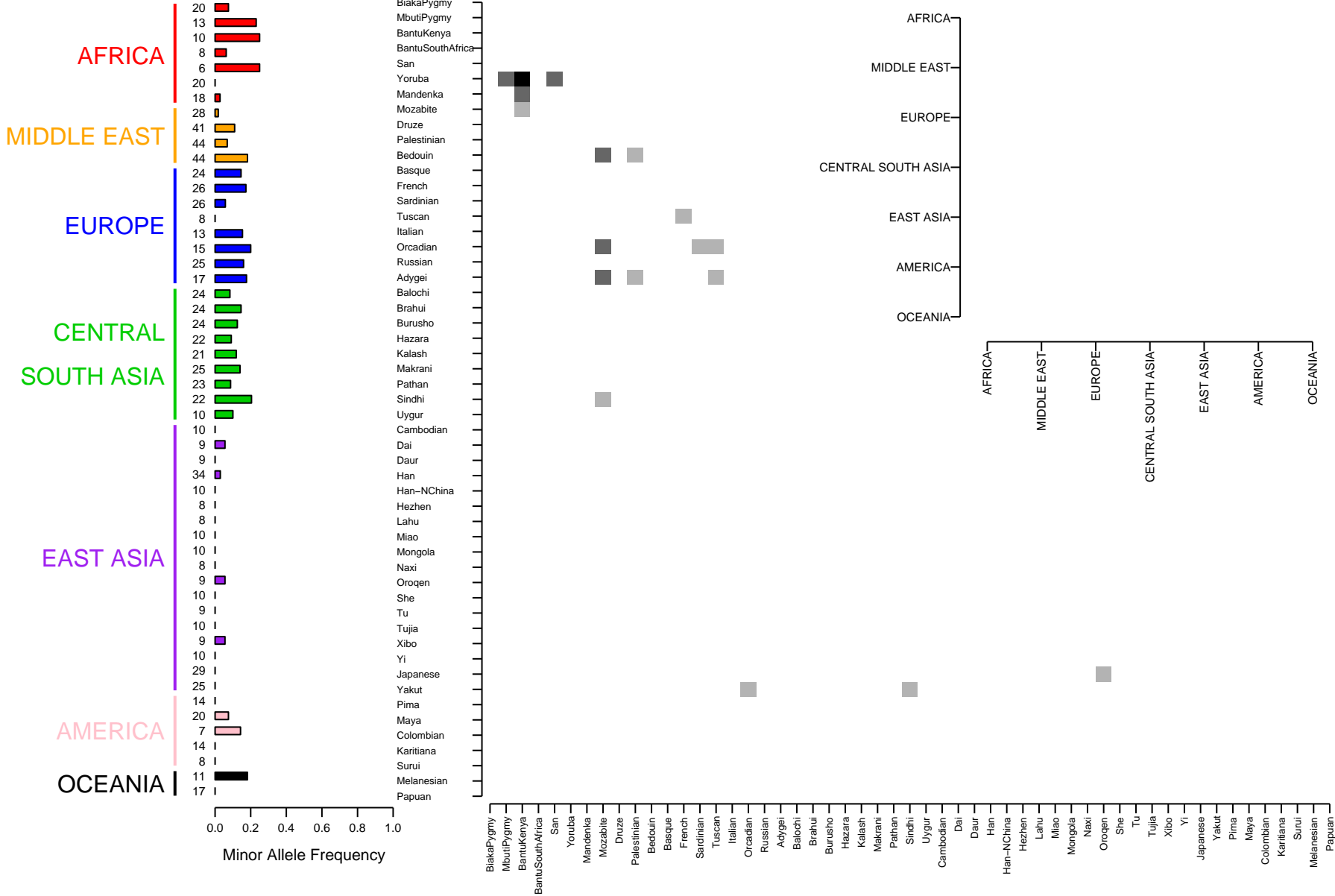

rs3760843

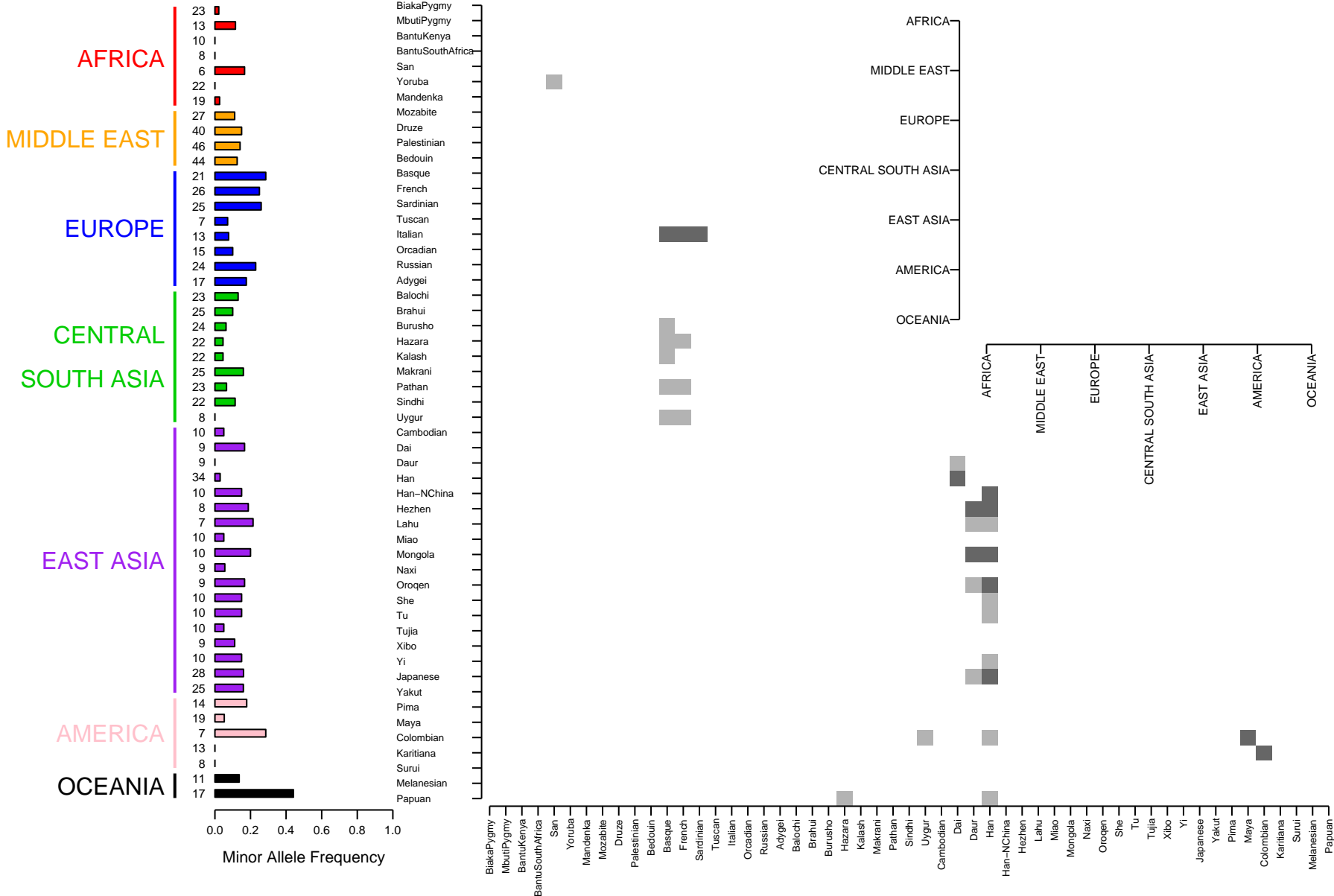

rs2143877

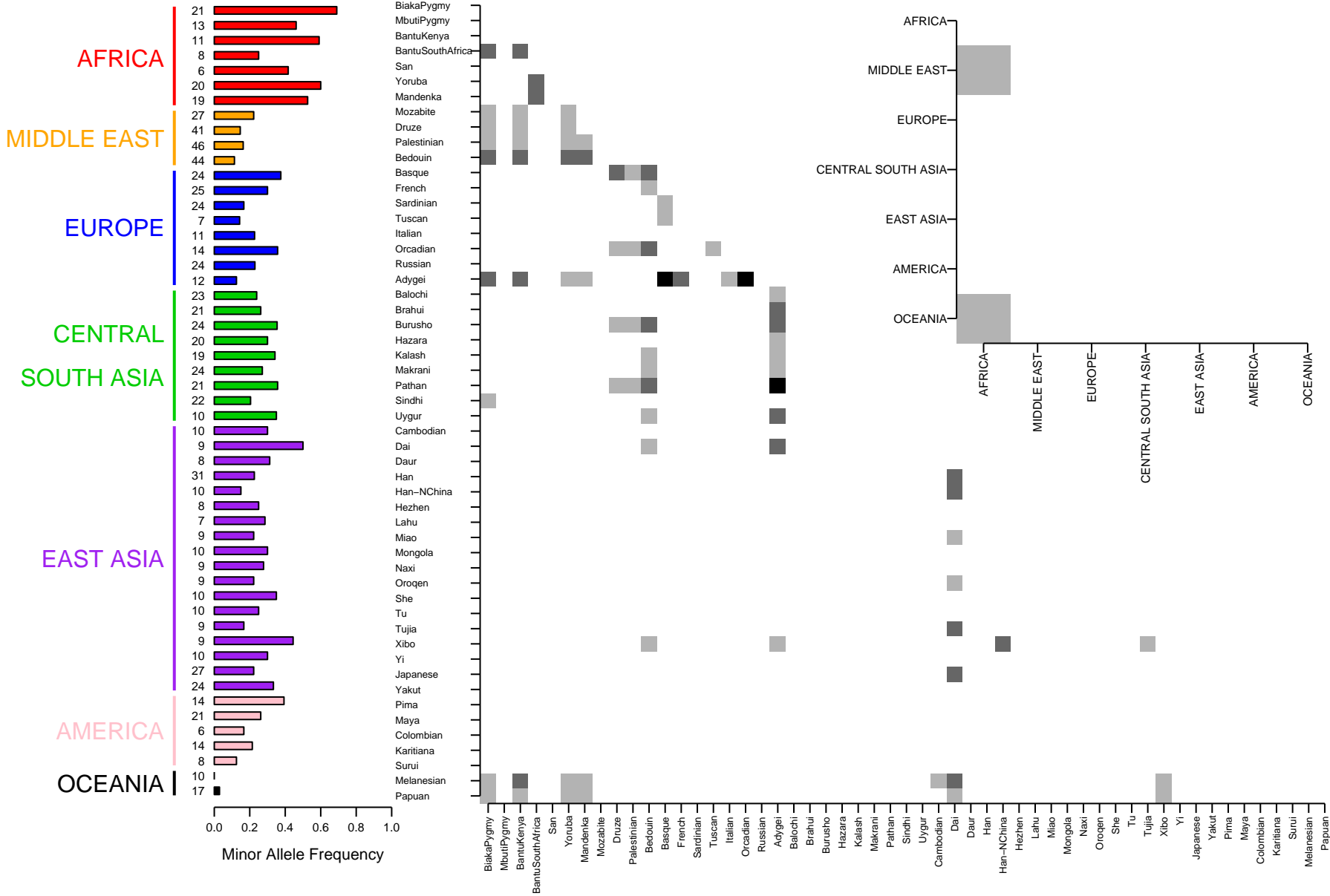

Supplement: Additional file 1 — Worldwide risk allele frequencies and population differentiation for the 10 BritAIMs. The dbSNP ID is found at the top of each figure. Minor allele frequencies are displayed in the vertical bar chart with sample size in number of individuals to the left. Each box in the 53 × 53 and 7 × 7 matrices represents a pairwise Fst comparison between populations and geographic regions, respectively. The shaded boxes in the matrices indicate which pairwise Fst values are significant compared to the empirical distribution at three P value thresholds (see the boxed-in P value legend of Figure 4). [file 1755-8794-2-45-S1.pdf]
